# The relationship between gratitude and adolescents’ prosocial behavior: A moderated mediation model

**DOI:** 10.3389/fpsyg.2022.1024312

**Published:** 2022-12-14

**Authors:** Ding Zhang

**Affiliations:** Center of Student Psychological Development, Zhoukou Normal University, Zhoukou, China

**Keywords:** gratitude, sense of meaning in life, self-esteem, prosocial behavior, adolescent

## Abstract

**Introduction:**

The development of prosocial behavior is an important embodiment of youth socialization. Cultivating adolescents’ prosocial behaviors can be beneficial to individuals form correct outlook on life and values, and has a profound impact on promoting the harmonious development of society.

**Methods:**

This manuscript constructs a model to explain the mediating role of sense of meaning in life between gratitude and prosocial behavior and the moderating role of self-esteem in the mediating path. From the analysis of the data of 2,735 questionnaires utilizing SPSS 22.

**Results:**

(1) Gratitude has a significant positive effect on prosocial behavior; (2) sense of meaning in life plays a mediating influence on the relationship between gratitude and prosocial behavior; and (3) self-esteem has a moderating impact on relationship between sense of meaning in life and prosocial behavior, which means that adolescents with high self-esteem experience a stronger positive effect of a sense of life’s meaning on their prosocial behavior.

**Discussion:**

These findings not only add to research on the relationship between gratitude and prosocial behavior, but also provide ideas for improving adolescent prosocial behavior. Theoretical and practical implications, along with limitations and future research directions, were discussed.

## Introduction

Prosocial behavior refers to the behavior that individuals consciously make to benefit others in social communication situations (Eisenberg et al., 1998). Prosocial behavior is one of the important manifestations of the development of adolescents’ social ability, and also an important yardstick of adolescents’ moral development (Jeffrey et al., 2008). It plays a key role in the development of individual mental health and socialization (Caprara et al., 2012). Adolescents are in a critical period of character formation and behavioral development. It is of great significance for the healthy growth of adolescents to explore the factors affecting their prosocial behaviors.

Although researchers have not yet reached a consensus on the concept of gratitude, there are still two tendencies: one is that gratitude is a personality trait (Peterson, 2017); the other suggests that gratitude is an emotional state (McCullough et al., 2004). However, these two points of view both prove that gratitude can promote individual prosocial behavior. McCullough et al. (2001) believes that gratitude is conceptualized as a moral affect that is analogous to other moral emotions such as empathy and guilt and he proposed the theory of moral affect. This theory illustrates that gratitude is an emotional response to the moral behavior of others and takes an important role in people’s moral life. Based on this theory, Chinese researcher He et al. (2012) proposed the three-dimensional structure model theory of gratitude. He believes that when people become the beneficiaries of prosocial behavior, they will feel grateful. Taking gratitude as a moral emotion means that gratitude comes from and can stimulate moral behavior. Gratitude comes from observing that the benefits you get are given by others, and realizing in your heart that this benefit is not due to you, which causes individuals to have moral motives and make prosocial behaviors to repay the benefits they get.

Several studies in western countries have demonstrated certain mediating variables between gratitude and prosocial behavior. For example, social support was found to play a mediating role between gratitude and prosocial behavior (You et al., 2022). However, all these studies are based on the sociological perspective. Meaning-making model believes that the acquisition of the sense of meaning in life is a complex process, which is affected by situational stimuli, comparison, evaluation, judgment, and other processes, as well as individual cognition, emotion, and behavior (Park, 2010). Therefore, to clarify the development mechanism of prosocial behavior, it is necessary to employ the perspective of multifactor integration to investigate the mediation and moderating of prosocial behavior by using factors such as gratitude, sense of meaning in life and self-esteem.

Previous studies have explored the impact of sense of meaning in life on individual behavior, and found that different levels of sense of meaning in life have different effects on individual behavior. For example, after disaster events (earthquakes), people usually exhibit more prosocial behaviors (Li et al., 2013). This can be attributed to the fact that when people are threatened by the meaning of life, they may reconstruct the meaning of life by choosing compensation mechanisms and producing prosocial behaviors. In the oriental culture that emphasizes collectivism, people’s feelings, cognition, and expression of gratitude and prosocial behavior are different from those of western individualism. However, few empirical studies have been conducted on the role of sense of meaning in life in the relationship between gratitude and prosocial behavior in the previous literatures. Therefore, the impact of sense of meaning in life on prosocial behavior is worth investigating and vital important.

According to the buffer hypothesis of self-esteem, the buffer function of high self-esteem can improve the individual’s ability of regulating (Cast and Burke, 2002). As an important component of self-concept, self-esteem is an important aspect of individual mental health. High self-esteem can play a buffer role when individuals face the threat of life value (Rosenberg, 1965). Previous studies have demonstrated that individuals with high self-esteem evaluate themselves more positively and believe in their ability, so they are more prone to help others; however, individuals with low self-esteem possess a more negative evaluation of themselves, believing that they face more obstacles, which will prevent them from showing prosocial behavior (Moscardino et al., 2020). Although previous studies have revealed the impact of self-esteem on prosocial behavior. However, few studies have investigated the moderating effect of self-esteem on sense of life meaning and prosocial behavior. Therefore, it is necessary to explore the role of self-esteem in the generation mechanism of adolescent prosocial behavior.

To sum up, there is still a certain gap in understanding the impact mechanism of gratitude and prosocial behavior among the Chinese adolescents. This study was to investigate the influence of gratitude on adolescents’ prosocial behavior, the mediating role of sense of meaning in life and the moderating role of self-esteem, with adolescents aged 11–18 years as the subjects. This is of great significance to enhance the level of gratitude and prosocial behavior of the Chinese adolescents.

### Gratitude and prosocial behavior

According to the theory of moral emotion, gratitude can stimulate the inner prosocial behavior of individuals. In addition, it also has a reinforcing effect, that is, when individuals express gratitude, they will make the benefactor do more prosocial behaviors (McCullough et al., 2004). Research shows that individuals who get help and have gratitude are more likely to show prosocial behavior to the benefactor or others later (Oliveira et al., 2021). After receiving the help or concern of the benefactor, the beneficiary expresses his gratitude and realizes that a better world needs more contributions from himself or others. Therefore, individuals with gratitude tendency are more likely to show prosocial behavior. Gratitude is an effective way for people to achieve peace and tranquility after a long life, and also an effective way to make personal relationships harmonious (Emmons and McCullough, 2003). After receiving help from others, most grateful individuals will help the benefactor or even the stranger (Graham, 1988). Pang et al. (2022) showed that the higher the level of gratitude, the easier it is to practice positive helping behaviors. An experiment of stimulating the state of gratitude of the subjects also shows that the subjects tend to help the benefactor and others (Shoshani et al., 2021). These studies show that people who have gratitude are more likely to engage in prosocial behaviors. Based on these, we have established our first hypothesis:

**Hypothesis 1:** Gratitude can significantly positively predict adolescents’ prosocial behavior.

### The mediating role of sense of meaning in life

The sense of meaning in life is an important experience of life. Acquiring and maintaining the sense of meaning is one of the basic motivations of human beings, and is also a key factor affecting individual mental health (Van Tongeren and Green, 2010). Steger (2009) argued that the sense of meaning in life refers to people’s understanding of the meaning of life and their awareness of life goals, tasks, or missions. Generally speaking, individuals with higher sense of life experience higher levels of physical and mental health (Boyle et al., 2010), whereas loss of sense of life will have a negative impact on individuals, such as suicide, anti-social behavior (Littman-Ovadia and Steger, 2010). Research showed that the positive mental state of individuals is the main condition for obtaining the meaning of life. Hicks et al. (2012) stimulated subjects’ positive experience through experimental research and found that individual positive experience could significantly improve the individual’s sense of meaning in life experience. Fu et al. (2022) showed that gratitude affects individual prosocial behavior, positive mood, and life satisfaction. Individuals with a high level of gratitude tend to have a stronger ability to perceive positive emotions and actively explore the meaning of life in their social environment.

In addition, some investigations suggested that the meaning of life has a motivational role, which affects the current behavior choices of individuals (Yang et al., 2016). The model of meaning seeking proposes that meaning oriented coping has a positive role in improving altruistic awareness (Heine et al., 2006). Tremendous researches have shown that individuals with a high sense of meaning are more likely to practice prosocial behavior with positive life experiences (Li et al., 2019). Research shows that the sense of meaning in life positively predicts prosocial behavior. That is, individuals with higher sense of life will have more prosocial behaviors in real life (Wang et al., 2018).

On the basis of the fear management theory, having life meaning can avoid various realistic and potential threats (Pyszczynski et al., 2004). To pursue life meaning, individuals tend to engage in prosocial behaviors. Experimental studies have provided support. For example, subjects who have been induced to sense of life meaning (relative to the control group) have more prosocial tendencies (Van Tilburg and Igou, 2017). Baumeister et al. (2013) also proposed that people who attach importance to the meaning of life are more willing to take care of and help others as a contributor and are more likely to make beneficial contributions to the society than being a recipient. Based on these, we established our second hypothesis:

**Hypothesis 2:** The sense of meaning in life plays a mediating role in gratitude and prosocial behavior.

### The moderating role of self-esteem

Self-esteem is an individual’s evaluation of self-value, which refers to the individual’s specific evaluation of his own value and ability in specific areas. Not all people with a high sense of life have prosocial behaviors (Rosenberg, 1965). According to the construction model of life meaning, one of the sources of life meaning is self-evaluation. When the evaluation of oneself is low, it will bring great pressure to individuals, produce painful experiences, and affect individual behavior (Park, 2010). Previous studies have shown that people with high self-esteem have positive self-worth and a higher sense of life significance. Therefore, individuals with high self-esteem tend to confirm their value and show positive thinking about the meaning of life (Moscardino et al., 2020; Tan et al., 2021). Relevant studies have also confirmed that self-esteem plays a regulatory role between individual positive psychological factors and prosocial behaviors (Hu and Ding, 2011). These studies show that for individuals with different levels of self-esteem, their sense of meaning in life has different effects on prosocial behavior. Individuals with high self-esteem are more likely to have positive thinking about the meaning of life and lend a helping hand when others are in danger, so they show more prosocial behaviors; individuals with low self-esteem are more difficult to think about the positive significance of life. In order to avoid taking responsibility, they are not easy to perceive the needs of others and show less prosocial behavior. Based on these, we established our third hypothesis:

**Hypothesis 3:** Self-esteem plays a moderating role in the second half of the mediating path.

**FIGURE 1:**
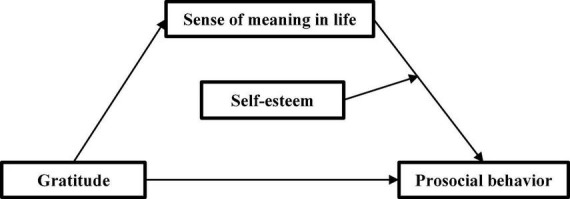
Research hypothesis model.

In summary, this study constructed a model (as shown in Figure 1) to explore the mediating and moderating mechanism of gratitude predicting the prosocial behavior, to provide ideas for improving adolescents’ prosocial behavior and promoting their healthy growth. Three hypotheses were put forward: (1) gratitude can significantly positively predict adolescents’ prosocial behavior; (2) the sense of meaning in life takes a mediating role in gratitude and prosocial behavior; (3) self-esteem plays a moderating role in the second half of the mediating path.

## Materials and methods

### Participants

A total of 2,850 students from two primary schools and two middle schools in Henan Province, China, were selected by cluster sampling. After excluding invalid questionnaires with excessively regular or missing answers, 2,735 valid questionnaires were collected, with an effective rate of 95.9%. There are 324 senior students in primary schools, 986 seventh grade students in two middle schools, 800 eighth grade students, and 625 ninth grade students. In the valid sample, 1,848 boys and 1,487 girls; 291 only children and 2,444 non-only children; 599 people from urban areas and 2,136 from rural areas; 544 student cadres and 2,191 non-student cadres. There are 1,973 fathers with high school education and below, and 762 fathers with high school education and above; 2,118 mothers with high school education or below, and 617 mothers with high school education or above; 1,077 people have left behind experience and 1,658 people have not (left behind experience refers to the experience that one or both parents leave their children for more than 1 year before the age of 16). Subjects were aged 10–18 years, with an average age of 13.96 years (SD = 1.55 years).

### Variables and measures

#### Adolescent gratitude scale

The adolescent gratitude scale (He et al., 2012), which contains 23 questions and is divided into six dimensions, is scored using five points, with a value of one indicating “completely inconsistent” and a value of five indicating “completely consistent.” The higher the score, the higher the gratitude level of the participants. The scale has good reliability and validity among Chinese teenagers (He et al., 2012). In this study, the Cronbach’s α coefficient of the scale was 0.81. This shows that the measures are reliable.

#### The meaning in life questionnaire

We use the sense of meaning in life scale compiled by Steger et al. (2006). The scale includes 10 questions on two dimensions: meaning experience and meaning seeking. Meaning experience measures whether an individual’s experience of life is meaningful, such as “I understand the meaning of my life.” Meaning seeking measures the degree of motivation of individuals to seek the meaning of life, such as “I am always looking for my own life goal.” Responses were recorded on a seven-point scale, with one indicating “completely inconsistent” and seven indicating “completely consistent.” Higher total scores correspond to a greater sense of meaning in life. Researchers tend to calculate the total score of the two dimensions to measure the level of life meaning, and test the applicability of the questionnaire under the Chinese cultural background, and find that the questionnaire has good reliability and validity in the use of Chinese teenagers (Wang et al., 2018). The Cronbach’s α coefficient of the scale was 0.71, indicating that the measures are reliable.

#### Self-esteem scale

The self-esteem scale developed by Rosenberg et al. (1995) consists of 10 items and was designed with the convenience of measurement in mind. Using a four-point scale, one represents “very good agreement” and four indicates “very bad agreement”; higher scores represent higher self-esteem. The scale has good reliability and validity among Chinese teenagers (Tan et al., 2021). In this study, the Cronbach’s α coefficient of the scale was 0.72, suggesting that the measures are reliable.

#### Prosocial tendencies measure

This research adopts the adolescent prosocial behavior tendency scale, which was compiled by Carlo and Randall (2002) and revised by Kou (2007). The scale has 26 questions and responses are scored on a five-point scale, with a value of one indicating “very unlike me” and a value of five indicating “very like me.” The scale contains six dimensions, with higher scores indicating a stronger prosocial behavior tendency. In the Chinese version of the scale, more researchers used the total score of six dimensions to judge the level of prosocial behavior, and tested the applicability of the questionnaire under the Chinese cultural background, and found that the questionnaire has good reliability and validity in the use of Chinese teenagers (Lei et al., 2022; Tian et al., 2022; Zhang et al., 2022). In this study, the Cronbach’s α coefficient of the scale was 0.74. This shows that the measures are reliable.

### Control variables

According to previous studies, in the context of school education, adolescents of different genders and ages have different levels of prosocial behavior (Tian et al., 2022; Zheng et al., 2022). Therefore, gender and age were used as control variables in the current study.

### Statistical treatment

After obtaining the informed consent of the school and the students, a written test was used in the class, and the instructions and confidentiality commitment were read by the trained instructor. SPSS version 22.0 (IBM, NY, United States) was used for the statistical analysis. Descriptive statistics were produced for all variables, while the PROCESS macro for SPSS (Model 4) was applied to examine the mediating effect of sense of meaning in life. Finally, the PROCESS macro for SPSS (Model 14) was used to examine the moderated mediating effect of self-esteem on the second half of the mediating path (Hayes, 2013). The significance of regression coefficient was tested by bootstrap method. The sample distribution constructs 5,000 samples through playback random sampling, and obtains the standard error and confidence interval of parameter estimation. In the current study, missing data were handled *via* the maximum likelihood estimates (ML).

### Data normal distribution detection and common method deviation control

The kurtosis coefficients of gratitude, prosocial behavior, sense of meaning of life, and self-esteem in this study were 1.2, 5.3, 3.5, and 2.7, respectively, which were less than 10. Their skewness coefficients were −0.56, 0.53, 0.94, and 0.33, with absolute values less than 3. It shows that the sample data is approximately normal distribution (Ghasemi and Zahediasl, 2012). As all the survey data were from the adolescent self-reports, there may be common method deviation. Therefore, the Harman single factor test was used to measure the deviation of variables. The results showed that the eigenvalues of 15 factors were greater than 1, and the explanatory power of the first factor was less than 40% of the critical value (the value of variation was 16.87%). Therefore, common method bias did not affect the data results.

## Results

### The mediating effect of sense of meaning in life and the moderating effect of self-esteem

A significant positive correlation was found among gratitude, sense of meaning in life, self-esteem, and adolescents’ prosocial behavior (Table 1).

**TABLE 1:** Mean, standard deviation, and correlation coefficient for each variable.

Variable	*M*	SD	1	2	3	4	5	6
Sex	0.54	0.49	–					
Age	13.96	1.55	0.06**	–				
Gratitude	92.50	12.73	0.12**	−0.21**	–			
Sense of meaning in life	51.45	10.71	–0.01	−0.16**	0.44**	–		
Self-esteem	28.21	4.77	–0.01	−0.13**	0.36**	0.36**	–	
Prosocial behavior	97.71	16.75	–0.01	−0.20**	0.52**	0.40**	0.23**	–

*N* = 2,735.

The bootstrap method was used for calculating the correlation coefficients. Gender is a dummy variable coded 1 for girls and 0 for boys, and the mean represents the proportion of girls.

**p < 0.01.

According to the method suggested by Hayes (2013), this study found that gratitude can predict prosocial behavior and mediate the sense of meaning in life. As Table 2 shows, in Equation 1, with prosocial behavior as the dependent variable, gratitude has a significant positive predictive effect on adolescent prosocial behavior (*c*_1_ = 0.52, *t* = 31.53, *p* < 0.001), and its total effect is significant. The interaction term of gratitude and self-esteem had a significant effect on adolescent prosocial behavior (*c*_3_ = 0.04, *t* = 2.73, *p* < 0.01), indicating that self-esteem had a significant moderating effect in the direct path. In Equation 2, with sense of meaning in life as the dependent variable, gratitude had a positive predictive effect on sense of meaning in life (*a*_1_ = 0.35, *t* = 19.67, *p* < 0.001).

**TABLE 2:** Test of moderated mediating model of gratitude on pro-juvenile prosociality.

Variable	Equation 1 (dependent variable: prosocial behavior)			Equation 2 (dependent variable: sense of meaning in life)			Equation 3 (dependent variable: prosocial behavior)		
	*b*	*t*	95% CI	*b*	*t*	95% CI	*b*	*t*	95% CI
Sex	–0.11	–3.07**	[–0.18, –0.04]	–0.11	–3.23**	[–0.17, –0.41]	–0.08	–2.76**	[–0.14, –0.02]
Age	–0.05	–4.12***	[–0.07, –0.02]	–0.05	–4.18***	[–0.07, –0.03]	–0.05	–4.53***	[–0.07, –0.03]
Gratitude	0.52	31.53***	[0.45, 0.57]	0.35	19.67***	[0.32, 0.39]	0.42	22.76***	[0.38, 0.46]
Self-esteem	0.23	12.10***	[0.01, 0.08]	0.24	13.40***	[0.20, 0.27]	–0.01	–0.36	[–0.04, 0.03]
Gratitude × Self-esteem	0.04	2.73**	[0.01, 0.07]	0.02	1.41	[–0.01, 0.05]	–	–	–
Sense of meaning in life							0.20	11.06***	[0.17, 0.24]
Sense of meaning in life × Self-esteem							0.04	2.52**	[0.01, 0.07]
*R* ^2^		0.27			0.20			0.31	
*F*		337.88***			225.05***			206.01***	

*N* = 2,735.

The bootstrap method was used for calculating the correlation coefficients. Gender is a dummy variable coded 1 for girls and 0 for boys, and the mean represents the proportion of girls.

**p < 0.01, ***p < 0.001.

In Equation 3, with prosocial behavior as the dependent variable, the sense of meaning in life had a significant effect on the prosocial behavior of adolescents (*b*_1_ = 0.20, *t* = 11.06, *p* < 0.001). This shows that the sense of meaning in life mediates the relationship between gratitude and prosocial behavior in adolescents. In addition, the interaction between sense of meaning in life and self-esteem had a significant impact on adolescents’ prosocial behavior (*b*_2_ = 0.04, *t* = 2.52, *p* < 0.05), signifying that self-esteem played a moderating role in the latter half of the mediation path. A1 and B2 were significant at the same time, suggesting that the moderated mediation model was established and the research hypothesis was supported.

**FIGURE 2:**
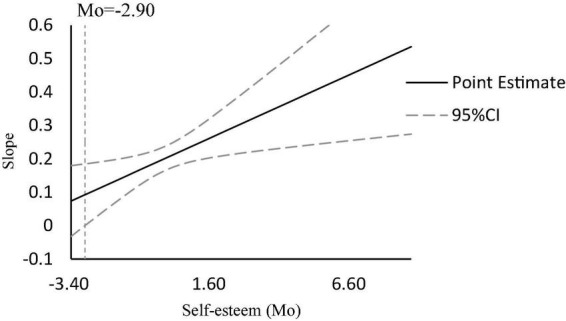
Moderating effects of self-esteem on the relationship between sense of meaning in life and prosocial behavior in adolescents.

In order to further reveal the moderating effect of self-esteem in the mediation path, Johnson–Neyman method was implemented to examine the moderating effect (Hayes, 2013). The results (Figure 2) show that when the moderator variable is less than −2.90, the slope is not significant; when the moderator variable is greater than −2.90, the slope is significant; and with the increase of self-esteem, the slope begins to be significant, and the higher the self-esteem level, the greater the slope.

## Discussion

Based on the theory of moral emotion, this study investigated the internal mechanism of prosocial behavior of Chinese adolescents. It confirmed the relationship between gratitude and prosocial behavior of Chinese teenagers, and provided support for the applicability of moral emotion theory in Chinese cultural environment. More importantly, different from previous studies, this study verified that the impact of sense of life on prosocial behavior is not absolute. When individual self-esteem is relatively high, it will accelerate the impact of sense of life on prosocial behavior. Conversely, when self-esteem is relatively low, it will inhibit the impact of sense of life on prosocial behavior. This study shows that self-esteem plays a complex role in the influence of gratitude on adolescents’ prosocial behaviors.

The results show that gratitude has a significant positive effect on adolescent prosocial behavior, and research hypothesis 1 has been verified. This is consistent with previous research results (Fu et al., 2022). At the same time, the results support Fredrickson’s (2001) theory that positive emotions can build individuals’ persistent mental resources. Individuals are driven to acquire and analyze resources more effectively, stimulate a positive and progressive way of thinking, and shape behavior in a positive way. Gratitude, as a positive emotional trait, can promote the production of personal positive behavior.

### The mediating role of the sense of meaning in life

This study found that the sense of meaning in life played a mediating role between gratitude and prosocial behavior. Positive emotional experiences are the core of the sense of meaning in life (Lin, 2022). Gratitude can promote the acquisition of positive emotional experiences and then form a strong sense of life’s meaning, which is consistent with previous studies (Unanue et al., 2022). Individuals with a high sense of meaning in life tend to have a clear understanding of themselves and can perceive the purpose and mission of their lives to continuously explore life’s value.

MacKenzie and Baumeister (2014) argued that the meaning of life can promote an individual’s awareness of specific event signals and help them recognize the behavior of others to acquire the positive factors and form a positive cognition of themself and others. Flouri and Sarmadi (2016) found that adolescents who participate in prosocial behaviors have good attribution preferences and positive information awareness. Therefore, individuals with a high sense of life’s meaning have more in-depth and positive thinking about their own life’s meaning and value, and a higher level of prosocial behavior. After the sense of meaning in life was added as a mediating variable in the model of this study, the effect of gratitude on the prosocial behavior of adolescents was still significant, indicating that gratitude plays a key role in these prosocial behaviors. Adolescence is the key period for the formation of good behavioral habits. In the process of receiving an education, it is necessary to continuously improve gratitude awareness. This involves developing a perspective in which one is grateful for society, nature, and other people, which then promotes the practicing of prosocial behaviors. This exemplifies the direct effect of gratitude on prosocial behavior as well as the indirect effect exerted through the sense of meaning in life.

### The moderating effect of self-esteem

Compared with previous researches on the relationship between gratitude and adolescents’ prosocial behavior, this study constructed a moderated mediation model to investigate the moderating effect of self-esteem on the mediating path of gratitude on adolescents’ prosocial behavior. This study found that the second half of the indirect effect of gratitude on prosocial behavior through the sense of life’s meaning was regulated by self-esteem. Specifically, compared with the group with high self-esteem, having low self-esteem weakened the relationship between the sense of life meaning and prosocial behavior. This result supports the fear coping theory of self-esteem, which states that self-esteem is a protective factor for individuals’ fear of life (Rosenblatt et al., 1989). When individuals with low self-esteem experience their own life’s meaning and value, they tend to experience more negative feelings and are not prone to prosocial behavior. In contrast, to maintain a positive evaluation of themselves, individuals with high self-esteem pay attention to their own needs and are often driven by autonomous motivation (Weinstein and Ryan, 2010), have more positive thinking about the meaning of life, have a clear understanding and pursuit of their own goals and objectives, and are more likely to produce prosocial behaviors.

## Conclusion

The current research has the following crucial theoretical and practical contributions. First, this study explored the impact of gratitude on adolescents’ prosocial behavior, and further clarified the mechanism of the sense of meaning in life and self-esteem. Secondly, the sense of meaning in life is a very important psychological factor in cultivating the prosocial behavior of young people. Finally, different self-esteem levels have different effects on prosocial behaviors. For school education, it is necessary to promote the improvement of adolescents’ self-esteem, stimulate them to develop more prosocial behaviors, and build and develop harmonious interpersonal relationships.

In summary, this study is an important step forward in understanding how gratitude relates to the prosocial behavior of Chinese adolescents. In China’s education, it is of great significance to improve the level of gratitude of young people and strengthen their prosocial behavior. First of all, it reveals the relationship between gratitude and youth in China. Secondly, the sense of life significance plays an intermediary role between gratitude and prosocial behavior, highlighting the important role of life significance in gratitude and prosocial behavior. In addition, the relationship between sense of meaning in life and prosocial behavior is regulated by self-esteem. In comparison with adolescents with low self-esteem, high self-esteem can enhance the impact of sense of life meaning on prosocial behavior.

Several limitations need to be considered when interpreting the findings. First, this study uses cross-sectional data, which cannot infer the causal relationship between variables. Therefore, it is necessary to use longitudinal designs to obtain stronger empirical evidence of causal evidence in future research. Second, this study only considered the positive effects of the sense of meaning in life and ignored the negative effects of some situational factors (such as, the sense of meaning in life is cultural, and the oriental culture pays more attention to the importance of face to self, thus affecting the individual’s self-evaluation) (Wang and Yang, 2005; Baumeister et al., 2013). Therefore, future research should focus on the impact of cultural factors on individual sense of meaning in life, so as to better explore the particularity of sense of meaning in life experience and better understand its essence, and increase the discussion on the meaning of life of local culture. Finally, the variability of high self-esteem is not considered, that is, whether the individual shows fragile high self-esteem or safe high self-esteem (Yang and Chen, 2020). Future studies will further refine the relationship between variables and consider the heterogeneity of high self-esteem.

## Data availability statement

The original contributions presented in this study are included in the article/supplementary material, further inquiries can be directed to the corresponding author.

## Ethics statement

The studies involving human participants were reviewed and approved by the Ethics Committee of Zhoukou Normal University. Written informed consent to participate in this study was provided by the participants’ legal guardian/next of kin.

## Author contributions

DZ independently completed the concept and design of the study, organized the database and statistical analysis, wrote the manuscript, and approved the submitted version.
